# TRAIL/NF-κB/CX3CL1 Mediated Onco-Immuno Crosstalk Leading to TRAIL Resistance of Pancreatic Cancer Cell Lines

**DOI:** 10.3390/ijms19061661

**Published:** 2018-06-04

**Authors:** Claudia Geismann, Wiebke Erhart, Frauke Grohmann, Stefan Schreiber, Günter Schneider, Heiner Schäfer, Alexander Arlt

**Affiliations:** 1 Laboratory of Molecular Gastroenterology & Hepatology, Department of Internal Medicine I, UKSH-Campus Kiel, 24105 Kiel, Germany; cgeismann@email.uni-kiel.de (C.G.); wiebke.erhart@uksh.de (W.E.); fraukethun@gmx.de (F.G.); s.schreiber@mucosa.de (S.S.); hschaef@1med.uni-kiel.de (H.S.); 2 Institute of Clinical Molecular Biology, UKSH Campus Kiel, 24105 Kiel, Germany; 3 Technische Universität München, Klinikum Rechts der Isar, II. Medizinische Klinik, 81675 Munich, Germany; guenter.schneider@tum.de; 4 Institute of Experimental Cancer Research, UKSH Campus Kiel, 24105 Kiel, Germany

**Keywords:** apoptosis resistance, TRAIL, pancreatic cancer, NF-κB, CX3CL1

## Abstract

Pancreatic ductal adenocarcinoma (PDAC) is one of the most lethal malignant neoplasms and registers rising death rates in western countries. Due to its late detection in advanced stages, its extremely aggressive nature and the minimal effectiveness of currently available therapies, PDAC is a challenging problem in the clinical field. One characteristic of PDAC is a distinct desmoplasia consisting of fibroblasts, endothelial and immune cells as well as non-cellular components, contributing to therapy resistance. It is well established that the NF-κB signaling pathway controls inflammation, cancer progression and apoptosis resistance in PDAC. This study attempts to identify NF-κB target genes mediating therapy resistance of humane PDAC cell lines towards death ligand induced apoptosis. By using a genome wide unbiased approach the chemokine CX3CL1 was established as a central NF-κB target gene mediating therapy resistance. While no direct impact of CX3CL1 expression on cancer cell apoptosis was identified in co-culture assays it became apparent that CX3CL1 is acting in a paracrine fashion, leading to an increased recruitment of inflammatory cells. These inflammatory cells in turn mediate apoptosis resistance of PDAC cells. Therefore, our data dissect a bifunctional cross-signaling pathway in PDAC between tumor and immune cells giving rise to therapy resistance.

## 1. Introduction

Pancreatic ductal adenocarcinoma (PDAC) is the fourth leading cause of cancer related death in Western countries. Poor therapy efficiency due to late diagnostic capabilities and a fast developing chemoresistance is reflected in rising death rates and makes PDAC to a challenging problem in the biomedical and clinical field [1].

Inflammation plays a crucial role in initiation and progression of PDAC. Together with a pronounced desmoplastic reaction, consisting of fibroblasts, immune and endothelial cells as well as extracellular matrix proteins inflammatory reactions represent a characteristic hallmark of PDAC [2,3]. Recently, the interaction of attracted immune cells with cancer cells gained particular attention because cytokines secreted by immune cells seem to promote the initiation, propagation and metastasis of tumors [4]. Beyond that, a continuously increasing body of evidence shows that in addition to the desmoplastic cells also the cancer cells express a variety of cytokines to create a tumor-promoting microenvironment. Thus, pancreatic cancer cells secrete for instance IL-13 to stimulate tumor promoting macrophages [5] or the chemokine CCL20 which facilitates the attraction of immune cells providing resistance against death receptor ligand mediated apoptosis [6]. In addition, a third modality of cytokine mediated tumor promotion, cancer cells express cytokine receptors and foster via the binding of the corresponding ligand an autocrine signaling loop which promotes cancer cell growth [4].

Because of the poor efficacy of conventional chemotherapeutic drug regimens, death receptor ligands are under investigation for PDAC treatment. Initially, the TNF alpha related apoptosis inducing ligand (TRAIL) was shown to be a quite promising and effective anti-cancer agent, which induced in cancer cells an extrinsic apoptotic response while sparing most normal cells. However, subsequent studies, demonstrated a robust resistance in a multitude of cancer cells against TRAIL induced apoptosis, among them pancreatic cancer cell lines [7,8,9]. This resistance could be attributed on one hand to constitutively upregulated anti-apoptotic proteins like c-FLIP, Bcl-XL and XIAP [10] and on the other hand to apoptosis inhibiting signaling pathways like protein kinase (pK)-c or NF-κB [8]. In line with this, an enhanced NF-κB activation correlates with TRAIL resistance in colorectal or pancreatic cancer cell lines [11,12].

The NF-κB transcription factor family consists of five subunits, NF-κB1 (also called p50), NF-κB2 (also called p52), RelA (also called p65), RelB and c-Rel. While all subunits bear a Rel homology domain (RHD) to form heterodimers only RelA, RelB and c-Rel harbor a transactivation domain (TAD) to bind cognate DNA elements and activate target genes [13,14,15]. Until now, the NF-κB target genes that convey chemoresistance in PDAC either through death receptor signaling or in association with chemotherapeutic drugs, are rarely investigated. Some studies propose a crosstalk with other transcription factors (STAT3) and the classical inhibitors of apoptosis (c-FLIP, cIAP) are supposed to be involved [16,17,18,19], too.

Given the fact that the inducible but not constitutive NF-κB activity prevents death receptor mediated signaling in PDAC cells [14], the present study aimed to identify RelA target genes jointly responsible for TRAIL mediated apoptosis resistance in pancreatic cancer cells. Thus, we identified the cytokine CX3CL1 as a direct NF-κB (RelA) target gene, which attracts immune cells to the site of tumor and thereby modulating the resistance of the cancer cells through a paracrine signaling pathway.

## 2. Results

### 2.1. Chemokine CX3CL1 is a RelA Target Gene

**Figure 1:**
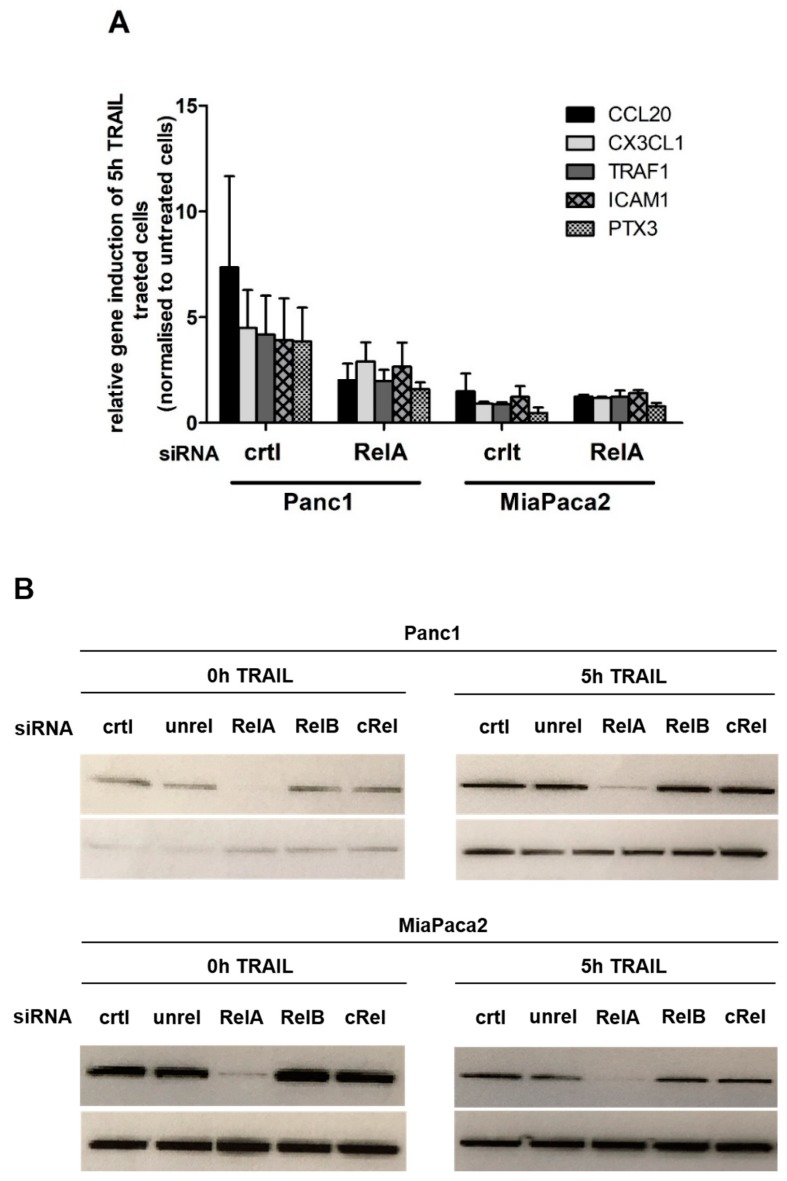
Top 5 upregulated TRAIL responsive genes in resistant PDAC cell lines. PDAC cells were transfected with the indicated siRNAs (unrel: unrelated control siRNA) for 48 h. Subsequent cells were left untreated or treated with 10 ng/mL TRAIL for 5h and a genome wide expression analysis (**A**) or western blot analysis (**B**) were performed. (**A**) Relative gene induction (5 h TRAIL treatment compared to untreated control siRNA cells) of the five strongest upregulated genes of the resistant cell line (Panc1) compared to the sensitive cell line (MiaPaca2) and the effect of transfection of the cells with RelA siRNA is shown. Mean of three independent replicates ± SD are shown; (**B**) Western bots whole cell lysates were analyzed using the p65/RelA antibody (**upper lane**) or Hsp90 antibody (**lower lane**) as control. One representative out of three is shown.

It is well established that an elevated NF-κB activity is associated with an augmented resistance against chemotherapy-induced apoptosis in pancreatic cancer [6,7,20]. To identify genes, responsible for the resistance towards TRAIL mediated apoptosis, genome wide transcriptome analysis with TRAIL resistant (Panc1) and sensitive (Miapaca2) PDAC cell lines were performed (series accession: GSE87287; http://www.ncbi.nlm.nih.gov/geo/query/acc.cgi?acc=GSE87287). In a second transcriptome analysis resistant Panc1 cells were first treated with specific siRNA towards the most abundant NF-κB subunit RelA or control siRNA and then treated with TRAIL to identify RelA dependent TRAIL target genes. Combining the results from both analysis, we were able to identify specific RelA target genes and candidates downstream thereof mediating the resistant phenotype (Figure 1A). Western blotting confirmed an effective reduction of the RelA subunit in untreated and 5 h TRAIL PDAC cells (Figure 1B). As expected, we observed no relevant change in protein levels of RelA through the TRAIL treatment alone.

The two strongest regulated genes identified by this procedure are two chemokines: CCL20 and CX3CL1. In this study, we will focus on the chemokine CX3CL1, because it is reported to be highly upregulated in PDAC specimens and elevated CX3CL1 expression in PDAC is associated with a poor prognosis in overall survival [21].

**Figure 2:**
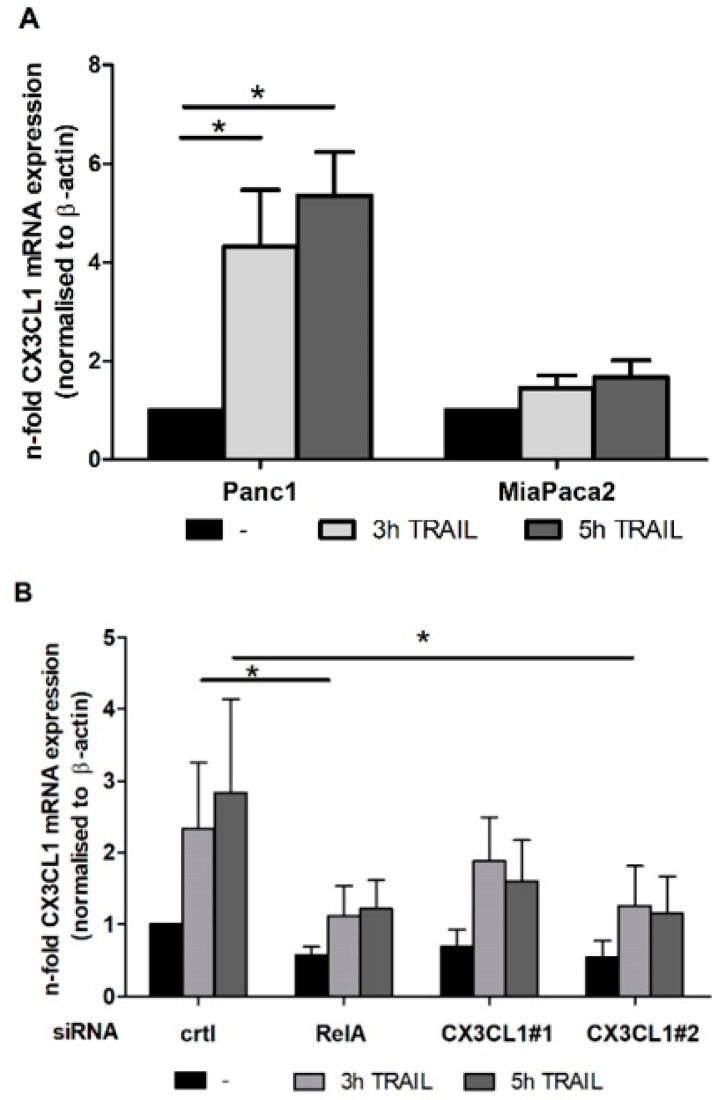

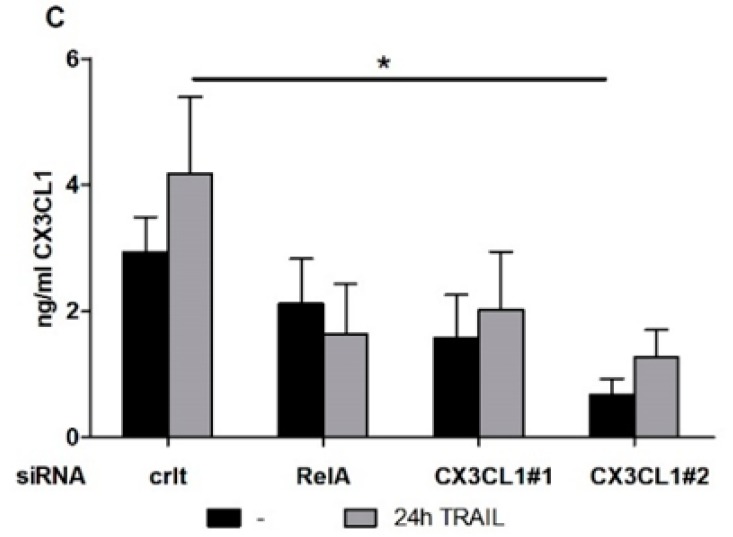
TRAIL upregulates CX3CL1 expression in resistant PDAC in a RelA dependent fashion (**A**) Panc1 and MiaPaca2 cells or (**B**) siRNA transfected Panc1 were treated TRAIL (10 ng/mL) for indicated times or left untreated and CX3CL1 mRNA expression was analyzed by realtime PCR. For control, β-actin qPCR was conducted; (**C**) Media were chanced to 0.5% FCS containing media 48 h after transfection and cells were treated with 10 ng/mL TRAIL for left untreated. After 24 h supernatants of Panc1 cells were collected and the concentration of CX3CL1 in the media was determined by ELISA. Data represent mean values ± SD from four independent experiments performed in duplicates. * *p*-values < 0.05.

In a first step, the results of the transcriptome arrays were verified. Panc1 and MiaPaca2 cells were treated with 10 ng/mL TRAIL for 3 and 5 h or were left untreated. As shown in Figure 2A, the CX3CL1 mRNA expression was significantly upregulated after 3 and 5 h of TRAIL treatment compared to untreated Panc1 cells. By contrast, in TRAIL sensitive MiaPaca2 cells the low basal CX3CL1 expression was almost unaffected by TRAIL treatment.

The knock down of the NF-κB subunit RelA with a specific siRNA led to a reduction of CX3CL1 mRNA expression (Figure 2B) and in accordance with this, both, the basal as well as the TRAIL induced protein expression were clearly reduced in RelA siRNA treated cell supernatants (Figure 2C). The sensitive MiaPaca2 cells expressed CX3CL1 protein below the detection limit of the ELISA. To enable the analysis of the function of CX3CL1 in TRAIL mediated apoptosis resistance in further experiments, the efficacy of two independent CX3CL1 specific siRNAs was confirmed on RNA (Figure 2B) and protein level (Figure 2C) in Panc1 cells.

### 2.2. RelA Binds to CX3CL1 Promoter after TRAIL Treatment

**Figure 3:**
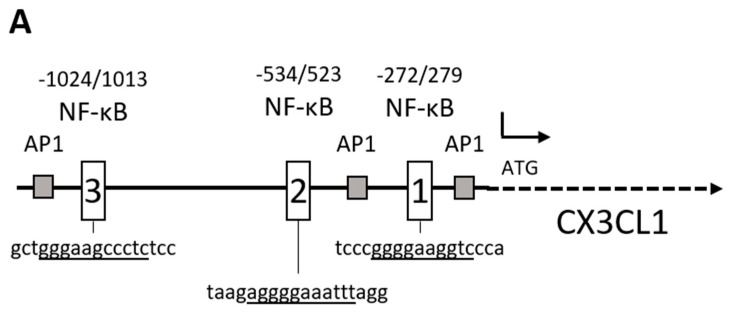

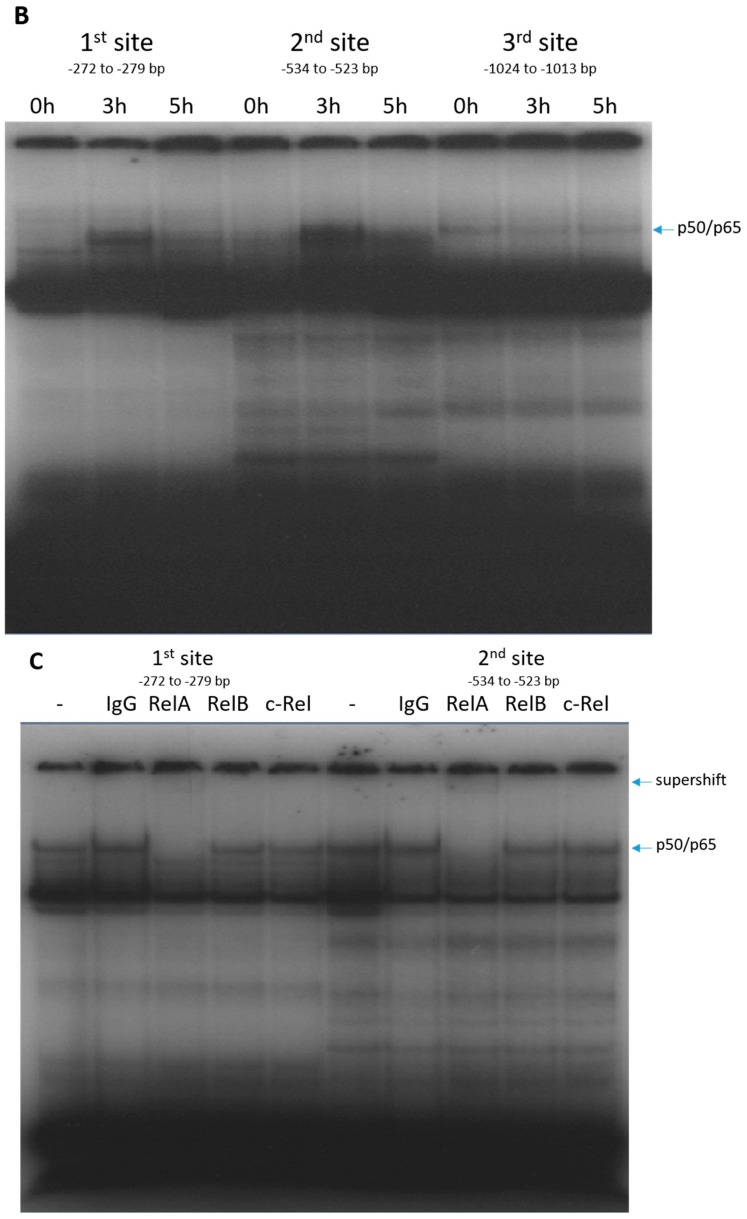
Binding of p50/RelA to putative NF-κB binding sites of the CX3CL1 promoter. (**A**) By sequence analysis three recognition motifs for NF-κB binding were identified in the CX3CL1 promoter. The consensus motif of the DNA sequences in these regions (written below) is underlined; (**B**) Panc1 cells were left untreated or treated with 10 ng/mL TRAIL for 3 h or 5 h, respectively. Nuclear extracts were submitted to EMSA with an oligonucleotide containing the potential NF-κB binding site of the CX3CL1 promoter. One representative out of two independent experiments is shown; (**C**) Nuclear extracts from 3 h TRAIL treated Panc1 cells were analyzed in supershift experiments using the indicated antibodies and oligonucleotides containing the potential NF-κB binding site of the CX3CL1 promoter. One representative out of two independent experiments is shown.

The CX3CL1 promoter harbors three putative NF-κB binding sites (Figure 3A and [22]) but, to our knowledge, none of these has been correlated with TRAIL induced CX3CL1 expression, yet. To show the binding of RelA to the CX3CL1 promoter, nuclear extracts of TRAIL treated or untreated Panc1 cells were subjected to electro mobility shift assays (EMSAs) by using three different oligonucleotides, each containing one of the potential NF-κB binding site. As shown in Figure 3B, only the first (−279 to −272 bp) and second (−534 to −523 bp upstream of the putative transcriptional start site) NF-κB binding sites show a transient activation (3 h) after TRAIL treatment (Figure 3B). In supershift experiments, antibodies against RelA, RelB and c-Rel confirmed the presence of a RelA containing NF-κB complex to these two binding sites of the CX3CL1 promoter. By contrast, no binding of RelB or c-Rel containing NF-κB dimers was detected (Figure 3C).

### 2.3. CX3CL1 Does Not Induce Direct Apoptosis in Panc1 Cells

**Figure 4:**
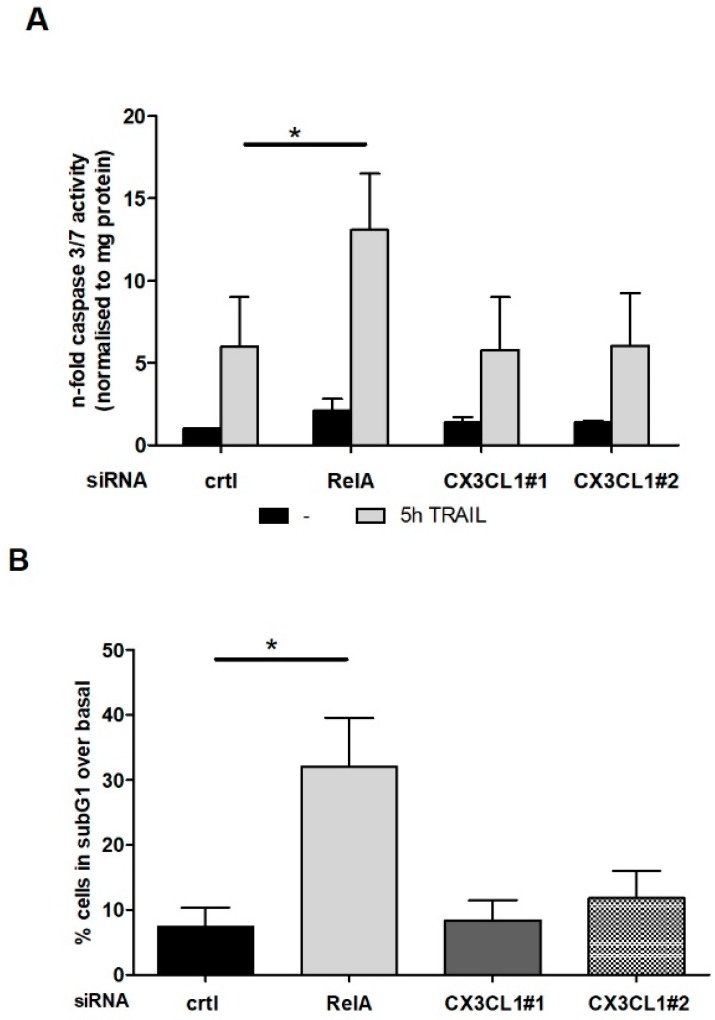
CX3CL1 does not directly affect TRAIL induced apoptosis in PDAC cells. Panc1 cells were subjected to CX3CL1 or RelA siRNA mediated knockdown for 48 h. After TRAIL treatment (10 ng/mL) apoptosis was measured by analyzing Caspase 3/7-activity 5 h after TRAIL stimulation (**A**) or subG1 fragmentation 24 h after TRAIL stimulation (**B**). Data of three independent experiments are expressed as n-fold Caspase 3/7-activity or % of subG1 content. Data represent mean values ± SD from three independent experiments performed in duplicates. * *p*-values < 0.05.

Pancreatic cancer cells make use of cytokines through autocrine-signaling pathways to elevate their own growth, angiogenesis or drug resistance [4]. This associates with an upregulated expression of the CX3CL1 receptor, CX3CR1, in pancreatic cancer specimen [23] and therefore our array analysis prompted us to investigate whether the TRAIL-NF-κB mediated CX3CL1 expression directly affects the apoptosis resistance of PDAC cells. Panc1 cells were treated with RelA or CX3CL1 specific siRNA, stimulated with TRAIL and analyzed for apoptotic cell death. Both caspase 3/7 assay and counting the subG1 fraction of cells showed no direct impact of CX3CL1 on the TRAIL mediated apoptosis while RelA specific siRNA led to a significant sensitization of the cells towards TRAIL induced apoptosis (Figure 4A,B) without significant effects of any siRNA on the basal apoptosis of untreated cells. Furthermore, we were able to exclude a direct effect of siRNA mediated knockdown of CX3CL1 on proliferation or migration of PDAC cells.

### 2.4. CX3CL1 Enhances the Migration of PBMCs towards PDAC Cells

**Figure 5:**
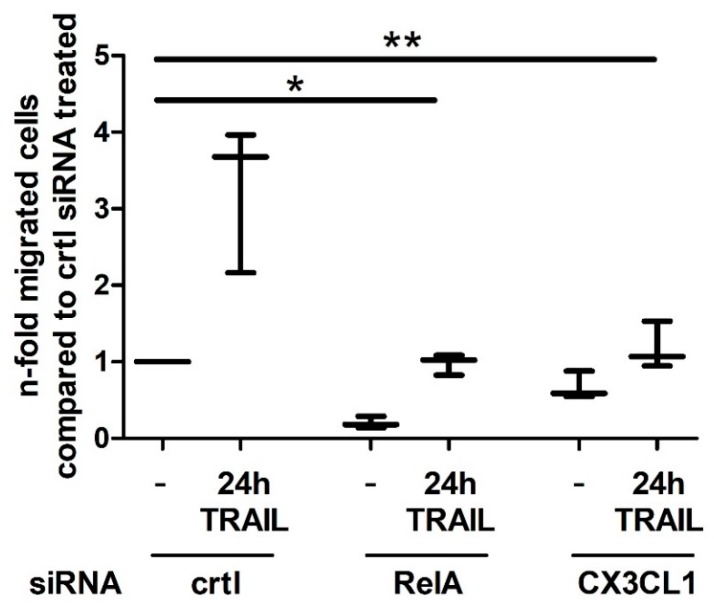
TRAIL induced PBMC migration is RelA and CX3CL1 dependent. PBMCs from healthy donors were isolated and subjected to migration assays. In a transwell system PBMCs were seeded into matrigel coated inserts and migration in dependence of the indicated Panc1 supernatants was measured. Plot from 3 independent measurements show the median (horizontal bar), the whiskers represent the minimum/ maximum data. * *p*-value < 0.025; ** *p*-value < 0.05.

The chemokine CX3CL1 acts as chemoattractant for macrophages, monocytes, dendritic cells, T-cells and natural killer cells, that express its receptor CX3CR1 [24,25]. Based on this, we investigated the migration of PBMCs from healthy volunteer donors in Boyden Chamber experiments as a function of the elevated CX3CL1 release in TRAIL treated PDAC cells. In approaches with supernatants from control siRNA transfected and 24 h TRAIL treated Panc1 cells, there was a significantly induced migration of PBMCs towards the lower compartment compared to supernatants from untreated cells (Figure 5). This enhanced TRAIL mediated migration of PBMCs was significantly reduced by the use of RelA and CX3CL1 specific siRNA, thus pointing to a TRAIL-RelA-CX3CL1 mediated attraction of immune cells.

### 2.5. CX3CL1 Attracted PBMCs Interfere with TRAIL Mediated Apoptosis in Panc1 Cells

Since we have shown that CX3CL1 has no direct, autocrine effect on PDAC cell apoptosis under TRAIL treatment and observed an enhanced migration of the PBMCs due to elevated CX3CL1 expression, we next analyzed the impact of CX3CL1 attracted immune cells on Panc1 cell apoptosis.

**Figure 6:**
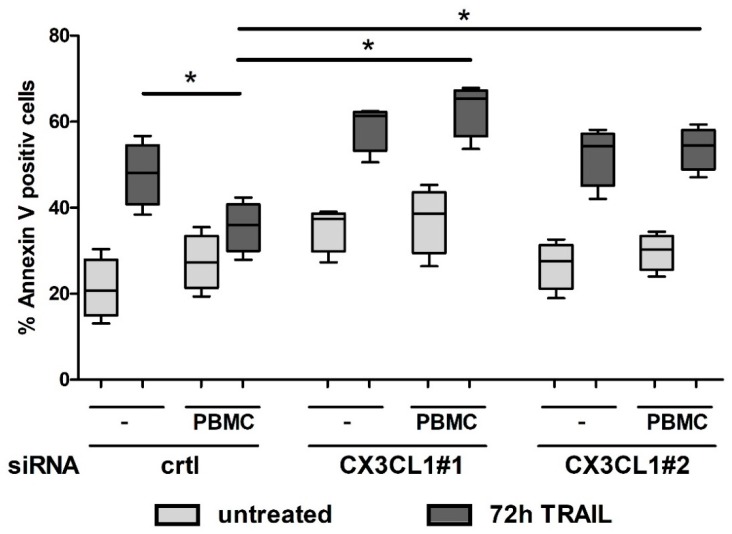
PBMC mediated increase of resistance of PDAC cells towards TRAIL treatment. Panc1 cells were transfected with indicated siRNAs, mono- or co-cultured with PBMCs for 72 h and treated with or without 10 ng/mL TRAIL. Co-cultured Panc1 cells were subjected to AnnexinV/PI apoptosis assay. Box plots from 4 independent measurements show the 25% quantile, the median and 75% quantile (horizontal bars), the whiskers represent the minimum/maximum data of n-fold AnnexinV positive cells * *p*-values < 0.05.

In co-culture experiments, siRNA treated Panc1 cells were cultured with PBMCs, TRAIL treated and subsequently subjected to apoptosis assay. As shown in control siRNA treated cells, the resistance towards TRAIL mediated apoptosis was further enhanced by the co-cultivation with PBMCs (Figure 6). Knocking down the CX3CL1 expression in Panc1 cells by specific siRNA abolished this TRAIL resistance inducing effect by the PBMC co-culture, thus supporting a paracrine role of CX3CL1 in apoptosis resistance of PDAC cells.

## 3. Discussion

To identify NF-κB target genes, especially RelA target genes responsible for resistance towards TRAIL induced apoptosis, a genome wide unbiased approach was applied and identified two chemokines as the most regulated genes: CX3CL1 and CCL20. Combining this result with the fact that chemokines lead to an elevated attraction of immune cells underlines the importance of an interplay of tumor and immune cells in cancer development and progression.

CX3CL1, originally named fractalkine, is a unique member of the forth class of chemokines (CX3C) and interacts with its highly selective chemokine receptor CX3CR1 which is expressed on different lymphocytes (NK-cell, T-cells, dendritic cells, monocytes) [26,27]. In smooth muscle cells, pro-inflammatory cytokines like TNF-α or IFN-γ induce CX3CL1 expression [28]. TRAIL resistant pancreatic cancer cell lines show a higher basal and inducible NF-κB activity compared to sensitive cell lines [6,7] and TRAIL treatment induces CX3CL1 expression in these cells. This inducible CX3CL1 expression was traced back to NF-κB binding to the −272/279 and −534/523 bp NF-κB binding sites of the CX3CL1 promoter, as shown by electromobility shift assays. This TRAIL mediated activation of CX3CL1 is driven by a RelA centered NF-κB signaling pathway, while the other transactivating subunits RelB and c-Rel had no impact on CX3CL1 expression. These results are in line with CX3CL1 promoter analysis in smooth muscle cells where a binding of the NF-κB subunit RelA to the CX3CL1 promoter during atherogenesis is shown. By luciferase assays, Gan et al. verified a functionally active region within 655 bp upstream of the transcriptional starting point in the CX3CL1 gene, but no further enhancement through an enlargement of the analyzed promoter fragment was observed [22].

CX3CL1 is a transmembrane chemokine expressed either on the cell surface or as a soluble glycoprotein, each mediating different biological activities. While the membrane-anchored CX3CL1 functions as an adhesion molecule fostering the retention of lymphocytes to CX3CL1 expressing endothelial cells the shed soluble form acts as a chemoattractant inducing migration and extravasation of lymphocytes into inflamed tissue [29]. In this context, the attraction of splenic lymphocytes by supernatants of CX3CL1 transduced murine hepatocellular carcinoma or neuroblastoma cells was shown [30,31]. Conditioned media from IFN-γ activated smooth muscle cells also induce a CX3CL1 mediated monocyte chemotaxis towards the increasing chemoattractant signal [22]. Visa versa, the transduced expression of receptor CX3CR1 in T lymphocytes led to an enhanced homing towards CX3CL1 producing tumors [27]. In line with these observations, our results show an augmented migration of PBMCs based on a TRAIL mediated activation of RelA/ NF-κB and subsequent CX3CL1 expression in pancreatic cancer cells.

Similar to the split function of CX3XL1 are clinical discrepancies reported on the role of CX3CL1/CX3CR1 axis in tumors. On the one hand, the CX3CL1/CX3CR1 signaling transduces antitumor effects going along with a better prognosis for the patient like in hepatocellular, gastric adeno carcinoma [32,33]. Ohta et al. attribute the better prognosis of colorectal cancer patients with high CX3CL1 expression to a recruitment of cytotoxic T-cells and NK cells which for their part mediate tumor cell cytotoxicity using a perforin and granzyme B mechanism [34]. On the other hand, CX3XL1/CX3CR1 signaling axis transduces pro-survival, proliferative and metastatic signals, favoring tumor progression. In breast and prostate cancer a higher expression of CX3CL1 is associated with the occurrence of metastasis [35,36]. Human surgical specimen from pancreatic cancer patients revealed a high expression of CX3CL1 compared to control tissue. This elevated CX3CL1 expression could be further attributed to significant shorter overall survival times compared to patients with low CX3CL1 levels [21]. The mechanisms, by which the CX3CL1/CX3CR1 axis affects the pathogenesis and survival of cancer cells, are poorly explored. In epithelial ovarian carcinoma specimen CX3CL1 expression was correlated with positive Ki67 proliferation marker staining and phosphorylation of Akt, whose hyperactivation is related to the control of cell proliferation [37]. The treatment of aortic smooth muscle cells with recombinant CX3CL1 protein promotes cell proliferation and the phosphorylation of Bad, inhibiting its apoptotic activity [28]. In the present study, we could not show a direct impact of CX3CL1 on TRAIL mediated apoptosis but provide instead strong evidence for a paracrine-signaling pathway which might contribute to the described protumorigenic action of CX3CL1 in pancreatic cancer cells. The PBCM-mediated resistance towards TRAIL was shown to be at least in part on a RelA-CX3CL1 paracrine pathway.

To further consolidate the described cancer cell-immune cell interplay as a potential point of action of anti- tumor approaches and to get a better understanding of the diverse functions of CX3CL1 in discrete tumor entities additional analysis have to be done. Nevertheless, our data underscore the relevance of a cancer-immune crosstalk for novel therapeutic interventions.

## 4. Materials and Methods

### 4.1. Materials

Cell culture media, supplements and fetal calf serum (FCS) were purchased from Biochrom (Berlin, Germany), human serum albumin (HSA) from Grifols (Frankfurt, Germany) and Killer-Trail was from Enzo Life Science/Alexis (Lörrach, Germany).

### 4.2. Cell Culture

The human PDAC cell line Panc1 was cultured in RPMI 1640 medium supplemented with 10% FCS, 1% l-glutamine and 1% sodium pyruvate. MiaPaca2 cells were cultured in Dulbecco’s Modified Eagle Medium (DMEM, high glucose) supplemented with 10% FCS, 2.5% HSA and 1% l-glutamine. The cells were incubated at 37 °C with 5% CO_2_ at 85% humidity. Both cell lines were obtained from Deutsche Sammlung von Mikroorganismen und Zellkulturen (DSMZ), Braunschweig.

### 4.3. RNA Preparation and Realtime PCR

Total-RNA preparation was performed by using RNAeasy Kit (Qiagen, Hilden, Germany) following the manufacturer’s instructions and 500 ng of RNA were applied to Reverse Transcription (Thermo Scientific, Whaltham, MA, USA). Realtime PCR was done by using QuantiNova SYBR Green PCR Kit (Qiagen) and a final concentration of 0.3 nM gene specific primer. Primers for mRNA expression were purchased from Eurofins Genomics (Ebersberg, Germany):

CX3CL1-F: 5′-CACCACGGTGTGACGAAATG -3′; CX3CL1-R: 5′-TCTCCAAGATGATTGCGCGT-3′; β-actin-F: 5′-CTCTTCCAGCCTTCCTTCCT-3′; β-actin-R: 5′-AGCACTGTGTTGGCGTACAG-3′.

### 4.4. siRNA Transfection

Panc1 cells were submitted to lipofection. In detail, cells were seeded in 12 well plates and transfection was done using 40 pMol control, RelA or CX3CL1 siRNA mixed with 12 µL HiPerFect transfection reagent (Qiagen). For each gene target at least two different siRNAs were used: RelA: s11915, s11916; RelB: si11917; cRel si11905; CX3CL1: s12630; s12631 (Thermo Scientific).

### 4.5. Gel Shift Assays

Preparation of nuclear extracts and gel shift assays were done as described previously [7]. For the detection of NF-κB binding to the CX3CL1 promoter three different γ^32^-P-labeled oligonucleotides (CX3CL1- NF-κB-1: 5′-CCAGCCTCCCGGGGAAGGTCCCAGTATGAC-3′; CX3CL1- NF-κB-2: 5′-GATTCTAAGAGGGGAAATTTAGGGGTCCAT; CX3CL1-NF-κB-3: 5′-GGCCGGTGCTGGGAAGCCCTCTCCCCATTG (MWG Eurofins) harboring an already described NF-κB recognition site (underlined) [22] were used. Supershift experiments were performed by inserting 4 µg of RelA, RelB or cRel antibody into the preparation (all Santa Cruz Biotechnology, Heidelberg, Germany).

### 4.6. Genome Wide Transcriptome Profiling and Cluster Analysis

All transcriptome data were collected and analyzed as described [6]. Processing was done according to MIAME standards and made publicly available by submitting to NCBI GEO (series accession: GSE87287; http://www.ncbi.nlm.nih.gov/geo/query/acc.cgi?acc=GSE87287).

### 4.7. PBMC Isolation

Isolation of PBMCs from healthy adult blood donors was performed as described before [6]. Isolated PBMCs were cultured in RPMI 1640 media containing 1% penicillin/streptomycin, 1% l-glutamine and 0.5% HSA.

### 4.8. Migration Assay

For analyzing the migration of PBMCs transwell inserts (5.0 µm pore size; 24 well, Costar, NY, USA) were coated with 100 µL of Matrigel (growth factor reduced, BD Bioscience, Heidelberg, Germany) diluted 1:5 with RPMI 1640 supplemented with 0.5% HSA, 1% l-glutamine, 1% penicillin/streptomycin. After coagulation 500 µL of conditioned supernatants were given into the wells of the plate and 50.000 PBMCs were seeded into each transwell. After 24 h inserts were discarded, supernatants were collected and migrated cells were counted by flow cytometry (BD-Facs Verse, BD-Bioscience, Heidelberg, Germany).

### 4.9. Co-Culture Assay

Previous to co-culture assay 30.000 PDAC cells were seeded into 24 well plates and transfected with the indicated siRNAs specified above. After 20 h media were replaced by 500 µL of x-Vivo15 cell culture media (Lonza, Cologne, Germany) ±10 ng/mL TRAIL. To guarantee the observed effects, based exclusively on paracrine signaling, cell culture inserts with a pore size of 0.4 µm (Greiner bio-one, Frickenhausen, Germany) were clipped in and 60.000 PBMCs were seeded into these inserts. Following 72 h of incubation at 37 °C, the PDAC cells were subjected to Annexin V/PI Assay.

### 4.10. ELISA

Panc1 cells were cultured as described above. Before stimulating the cells with 10 ng/mL TRAIL for 24 h, cell medium were substituted by RPMI 1640 supplemented with 1% l-glutamine and 1% sodium pyruvate. The culture supernatants were collected and centrifuged for 5 min at 1200 rpm and 4 °C. The cell free supernatants were aliquoted and frozen at −80 °C. Human CX3CL1 Quantikine ELISA from R&D (Wiesbaden, Germany) was done following the manufacturer’s instructions.

### 4.11. Western Blotting

For total cell lysate preparation cells were washed with PBS twice and harvested in lysis buffer (0.125 M Tris; 0.1% SDS, protease inhibitor cocktail). Gel electrophoresis and immuno-blotting were performed as already described [6] using RelA and HSP90 antibodies (both Santa Cruz Biotechnology).

### 4.12. Annexin V/PI Assay

Cells were trypsinized, washed with PBS and submitted to Annexin V-FITC Kit due to the manufacturer’s instructions (Miltenyi Bioscience, Bergisch Gladbach, Germany). Flow cytometry was carried out on a FACS Verse flow cytometer (BD Bioscience) and cells positive for Annexin V were regarded as being apoptotic.

### 4.13. Caspase-3/-7 Assay

Apoptosis induced by Killer-TRAIL was determined by the measurement of Caspase-3/-7 activity (Promega, Mannheim, Germany) according to the manufacturer’s instructions and as described [6]. All assays were done in duplicates. Caspase-3/-7 activity was normalized to the protein content of the analyzed cell lysates.

### 4.14. Statistics

Data represent the mean ± standard deviation and were analyzed by student’s *t*-test, *p*-values < 0.05 were considered as statistically significant and indicated by *.

## Abbreviations

PDAC	pancreatic ductal adenocarcinoma
TRAIL	TNF alpha related apoptosis inducing ligand
NF-κB	nuclear factor kappa-light-chain-enhancer of activated B cells
CX3CL1	chemokine (C-X3-C motif) ligand 1
CX3CR1	CX3C chemokine receptor 1
RelA	nuclear factor NF-kappa-B p65 subunit
